# Theoretical Investigations of the Hexagonal Germanium Carbonitride

**DOI:** 10.3390/ma11050655

**Published:** 2018-04-24

**Authors:** Xinhai Yu, Zhenyang Ma, Suriguge, Peng Wang

**Affiliations:** 1 College of Airworthiness, Civil Aviation University of China, Tianjin 300300, China; zym_airworthiness@163.com (Z.M.); pw_airworthiness@163.com (P.W.); 2 Department of Mechanical and Electrical Engineering, Hetao College, Bayannur Inner Mongolia 015000, China; suriguge87@163.com

**Keywords:** GeCN, mechanical properties, elastic anisotropy properties, electronic properties

## Abstract

The structural, mechanical, elastic anisotropic, and electronic properties of hexagonal germanium carbonitride (*h*-GeCN) are systematically investigated using the first-principle calculations method with the ultrasoft pseudopotential scheme in the frame of generalized gradient approximation in the present work. The *h*-GeCN are mechanically and dynamically stable, as proved by the elastic constants and phonon spectra, respectively. The *h*-GeCN is brittle because the ratio *B*/*G* and Poisson’s ratio *v* of the *h*-GeCN are less than 1.75 and 0.26, respectively. For *h*-GeCN, from brittleness to ductility, the transformation pressures are 5.56 GPa and 5.63 GPa for *B*/*G* and Poisson’s ratio *v*, respectively. The *h*-GeCN exhibits the greater elastic anisotropy in Young’s modulus and the sound velocities. In addition, the calculated band structure of *h*-GeCN reveals that there is no band gap for *h*-GeCN with the HSE06 hybrid functional, so the *h*-GeCN is metallic.

## 1. Introduction

Ternary compounds have attracted more and more attention, such as B–C–N [1,2,3], B–C–O [4,5,6,7] superhard materials, and Si–Ge–N [8,9], Si–C–N [10,11,12,13,14], Ge–C–N [15,16], and so on. Si–Ge–N is an alloy of silicon nitride and germanium nitride. The structural, elastic anisotropic, and electronic properties of *m-*Si_2_GeN_4_ and *m-*SiGe_2_N_4_ were investigated using density functional theory calculations by Ma et al. [8], where *m-*Si_2_GeN_4_ and *m-*SiGe_2_N_4_ are alloys of *m-*Si_3_N_4_ and *m-*Ge_3_N_4_. They found that the *m*-Si*_x_*Ge_3−*x*_N_4_ (*x* = 0, 1, 2, 3) series exhibit larger anisotropy and that the anisotropy of *m*-SiGe_2_N_4_ is largest among the *m*-Si*_x_*Ge_3−*x*_N_4_ (*x* = 0, 1, 2, 3). The calculated band structures show that both *m-*Si_2_GeN_4_ and *m-*SiGe_2_N_4_ are direct semiconductors with band gaps of 4.76 eV and 4.81 eV, respectively. Very recently, the structural, mechanical, anisotropic, electronic, and thermal properties of *t*-Si_2_GeN_4_ and *t*-SiGe_2_N_4_ in the tetragonal phase were systematically investigated by Han et al. [9]. They found that both *t*-Si_2_GeN_4_ and *t*-SiGe_2_N_4_ demonstrate brittleness, and that *t*-Si_2_GeN_4_ and *t*-SiGe_2_N_4_ exhibit larger elastic anisotropy than that of *c*-Si_2_GeN_4_ and *c*-SiGe_2_N_4_ characterized by Young’s modulus, Poisson’s ratio, the percentage of elastic anisotropy for shear modulus *A_G_*, the percentage of elastic anisotropy for bulk modulus *A_B_*, and the universal anisotropic index *A*^U^. The electronic structures of *t*-Si_2_GeN_4_ and *t*-SiGe_2_N_4_ are both wide-bandgap semiconductor materials, with band gaps of 3.94 eV and 3.83 eV using the HSE06 hybrid functional, respectively. In addition, the effects of temperature and pressure on the Debye temperature, thermal expansion coefficient, heat capacity, and Grüneisen parameters were discussed in detail utilizing the quasi-harmonic Debye model. In addition, other III–V group compounds have also been studied extensively, including three-dimensional materials [17,18,19,20,21,22] and some low-dimensional materials, such as few-layer *h*-AlN [23], buckled honeycomb XBi and XBi_3_ (X = B, Al, Ga, and In) sheets [24], and buckled III-Bi sheets [25].

Ternary Si−C−N compounds represent a series of novel functional materials that have attracted considerable research interest because of their excellent chemical and physical properties, which include good creep properties, high hardness, excellent oxidation resistance, and thermal shock resistance over a broad temperature range [26]. Several Si−C−N compounds with different chemical compositions have been synthesized through various approaches [27,28,29,30,31,32,33,34,35,36]. Very recently, Cui et al. [26] performed an extensive structural search of SiCN compounds using the crystal structure analysis by particle swarm optimization (CALYPSO) algorithm [37]. They revealed that the novel tetragonal SiCN (*t*-SiCN) was more energetically stable than the *c*-SiCN proposed 40 years ago [38], and two high-pressure phases of orthorhombic SiCN (*o*-SiCN) and hexagonal SiCN (*h*-SiCN) were also predicted in the work. The *h*-SiCN and *o*-SiCN were able to be quenched at ambient conditions and exist in metastable phases. The hardnesses of *t*-SiCN, *o*-SiCN, and *h*-SiCN were calculated to be 41.5, 30.0, and 30.2 GPa, respectively [26].

Recently, Xing et al. [15] first investigated the structural, mechanical, electronic, and thermodynamic properties of the tetragonal structure germanium carbonitride (*t*-GeCN) using the density function theory with the ultrasoft pseudopotential scheme in the frame of the generalized gradient approximation and the local density approximation. The elastic constants and phonon spectra have confirmed that *t*-GeCN is mechanically stable and that *t*-GeCN is dynamically stable. The electronic structure of *t*-GeCN shows that it is an indirect semiconductor with band gap of 0.63 eV. The anisotropy studies show that *t*-GeCN exhibits a larger anisotropy in its Poisson’s ratio, Young’s modulus, shear modulus, sound velocities, and universal elastic anisotropy index. However, the elastic anisotropy of *t*-GeCN in Young’s modulus is slightly smaller than that of hexagonal germanium carbonitride (*h*-GeCN).

The structure of *h*-GeCN is based on that of *h*-SiCN [26], with a germanium atom substituting for the silicon atom. The structural, mechanical, elastic anisotropy, and electronic properties of *h*-GeCN have not yet been studied. In this work, we have systematically investigated the physical properties of *h*-GeCN.

## 2. Theoretical Methods

The total energy calculations were performed using density functional theory (DFT) with the Perdew–Burke–Ernzerhof (PBE) exchange correlation in the framework of the generalized gradient approximation (GGA) [39] as implemented in the Cambridge Serial Total Energy Package (CASTEP) plane wave code [40]. The interactions between the ionic core and valence electrons were described by the ultrasoft pseudopotential [41], and the 2*s*^2^2*p*^2^, 2*s*^2^2*p*^3^, and 4*s*^2^4*p*^2^ were considered as valence electrons for C, N, and Ge, respectively. The equilibrium crystal structures were achieved by utilizing geometry optimization in the Broyden–Fletcher–Goldfarb–Shanno (BFGS) [42] minimization scheme. The plane wave basis set was truncated with a cutoff energy of 500 eV, and the Brillouin zone integration was generated using Monkhorst–Pack *k*-point meshes [43] with a high-quality grid of 0.025 Å^−1^ (8 × 15 × 9) for total energy and elastic constants calculations, respectively. The elastic constants were calculated by the strain–stress method, which has been successfully utilized previously [44,45]. The bulk modulus, shear modulus, Young’s modulus, and Poisson’s ratio were estimated via Voigt–Reuss–Hill approximation [46,47,48]. The HSE06 hybrid functional [49] was used for the calculation of the electronic structures of *h*-GeCN.

## 3. Results and Discussion

### 3.1. Structural Properties

**Figure 1:**
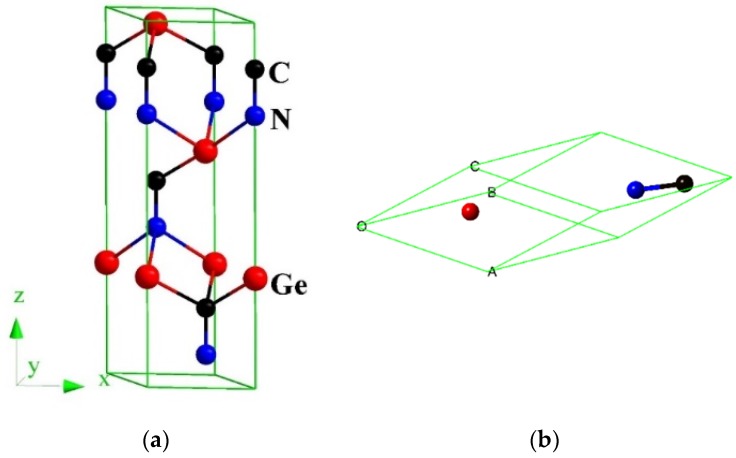
The crystal structures of *h*-GeCN: hexagonal representation (**a**) and rhombohedral representation (**b**).

**Figure 2:**
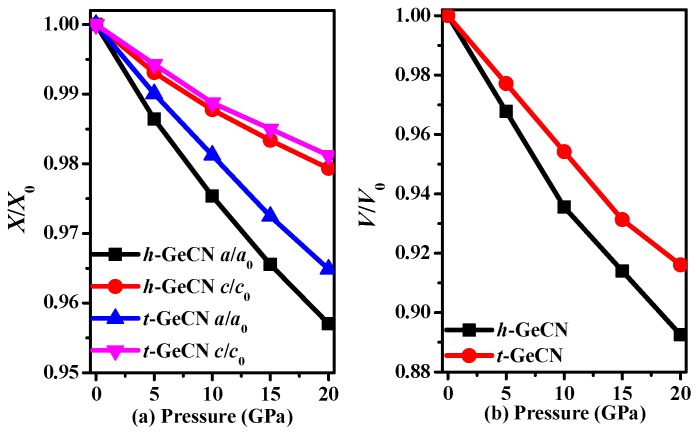
The lattice constants *X*/*X*_0_ (**a**) and primitive cell volume *V*/*V*_0_ (**b**) as functions of pressure for *h*-GeCN and *t*-GeCN.

The calculated lattice parameters of *h*-GeCN and *t*-GeCN, together with other theoretical results, are all listed in Table 1. For *t*-GeCN, the lattice parameters *a* and *c* of the GGA deviate from the corresponding previous values [15] by 0.07% and 0.13%, while lattice parameters *a* and *c* of the LDA (local density approximation) deviate from the corresponding previous values [15] by 0.26% and 1.47%; that is to say, the values of the GGA deviate from other previous values less than do those of the LDA. Therefore, in this paper, all the results are based on the GGA. In addition, the lattice parameters are *a* = 3.621 Å in this work, and *a* = 3.622 Å [2] with GGA for *c*-BN, while the lattice parameters are *a* = 3.582 Å in this work, and *a* = 3.576 Å [2] with LDA for *c*-BN; the experimental value of *c*-BN is 3.620 Å, so the result of GGA is very close to the experiment value for *c*-BN. Therefore, in this work, all the results are based on the crystal structure from GGA. The crystal structure of the hexagonal representation and rhombohedral representation for *h*-GeCN are shown in Figure 1. The red, black, and blue spheres represent Ge, C, and N atoms, respectively. For *h*-GeCN, the bond lengths of the C–N, C–Ge, and N–Ge bonds are 1.362 Å, 2.091 Å, and 2.180 Å, respectively. The C–N and C–Ge bond lengths are slightly greater than the N–Si (1.895 Å) and C–Si (1.875 Å) bond lengths in *h*-SiCN, while the C–N bond length is slightly smaller than that of C–N (1.373 Å) in *h*-SiCN. Compared with *t*-GeCN, the C–N bond length in *h*-GeCN is slightly smaller than that (1.445 Å) in *t*-GeCN, while the C–Ge and N–Ge bond lengths in *h*-GeCN are slightly greater than the C–Ge (2.015 Å) and N–Ge (1.884 Å) bond lengths in *t*-GeCN. In addition, there are C–C bond lengths (1.619 Å) in *t*-GeCN. The lattice constants and conventional cell volumes of *h*-GeCN and *t*-GeCN are shown in Figure 2. From Figure 2a, the compression along the lattice constants’ *a*-axis and *c*-axis for *h*-GeCN is slightly larger than that of *t*-GeCN when the pressure increases. In addition, it is clear that the compression of *h*-GeCN is slightly larger than that of *t*-GeCN; that is to say, the bulk modulus of *t*-GeCN is slightly larger than that of *h*-GeCN.

### 3.2. Stability and Mechanical Properties

**Figure 3:**
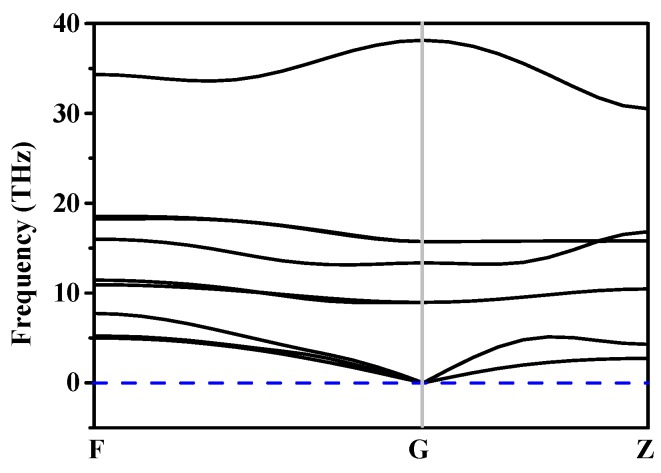
The phonon spectra for *h*-GeCN.

The stability of *h*-GeCN can be characterized by phonon spectra and Born stability conditions. The phonon spectra of *h*-GeCN are displayed in Figure 3. The phonon spectra show that all the lattice vibrations in the Brillouin region are positive, indicating that the *h*-GeCN is dynamically stable. The elastic constants of *h*-GeCN under different pressures are listed in Table 2. The criteria for mechanical stability of hexagonal symmetry are [50] *C*_44_ > 0, $C_{11}^{2}$ > $C_{12}^{2}$, and (*C*_11_ + 2*C*_12_)*C*_33_ > 2 $C_{12}^{2}$. From Table 2, we note that all the elastic constants of *h*-GeCN under different pressures satisfy the Born stability conditions of hexagonal symmetry.

The elastic moduli of *h*-GeCN under different pressures are listed in Table 3. According to our previous prediction, the bulk modulus of *t*-GeCN (183 GPa) is indeed larger than that of *h*-GeCN (130 GPa). Similarly, the shear modulus and the Young’s modulus are the same as the bulk modulus. However, the bulk modulus, shear modulus, and Young’s modulus of *h*-GeCN are all slightly larger than those of *m*-Ge_3_N_4_ [8]. A kind of material showing brittleness or ductility is usually characterized by two physical quantities: *B*/*G* and Poisson’s ratio *v*. A larger *B*/*G* [51] value (*B*/*G* > 1.75) and a larger *v* (*v* > 0.26) [52] for a solid represent a ductile state, while a smaller *B*/*G* value and a smaller *v* usually mean that the solid is brittle. The *B*/*G* and Poisson’s ratio *v* of *h*-GeCN are also presented in Table 3. From Table 3, with increasing pressure, both *B*/*G* and Poisson’s ratio *v* increase. At ambient pressure, *B*/*G* = 1.69 and *v* = 0.25 of *h*-GeCN, indicating that the *h*-GeCN exhibits brittleness. As the pressure increases, the *h*-GeCN changes from brittle to ductile. From brittleness to ductility, we note that the transformation pressures of *h*-GeCN are 5.56 GPa and 5.63 GPa for *B*/*G* and Poisson’s ratio *v*, respectively.

The Debye temperature (*Θ*_D_) is a fundamental physical property and correlates with many physical properties of solids, such as specific heat and the thermal coefficient [53]. *Θ*_D_ = (*h*/*k*_B_)[(3*n*/4π)(*N*_A_*ρ*/*M*)]^1/3^*v_m_*, where *h* is Planck’s constant; *k*_B_ is Boltzmann’s constant; *N*_A_ is Avogadro’s number; *n* is the number of atoms in the molecule; *M* is molecular weight; *ρ* is the density; and *v_m_* is the mean sound velocity, *v_m_* = [(2/$v_{s}^{3}$ + 1/$v_{p}^{3}$)/3]^−1/3^. The *v_l_* and *v_t_* are the longitudinal and transverse sound velocities, respectively, which can be obtained from Navier’s equation [54]: *v_p_* = [(*B* + 4*G*/3)/*ρ*]^1/2^, *v_s_* = (*G*/*ρ*)^1/2^. The calculated Debye temperature and sound velocity of *h*-GeCN under different pressures are listed in Table 4. At ambient pressure, the Debye temperature of *h*-GeCN is 506 K—smaller than that of *t*-GeCN (756 K). The Debye temperature of *h*-GeCN increases with increasing pressure except for the situation under 20 GPa. The changes of almost all of the sound velocities for *h*-GeCN are consistent with the changes of the Debye temperature, except for *v_p_*. The sound velocity *v_p_* increases with increasing pressure until the pressure increases to 20 GPa. The Debye temperature of *h*-GeCN shows different behavior at 20 GPa because the elastic constants and elastic moduli of *h*-GeCN decreased quickly from 15 to 20 GPa than from 10 to 15 GPa. Therefore, the Debye temperature of *h*-GeCN shows different behaviors at 20 GPa.

### 3.3. Elastic Anisotropy Properties

The sound velocities are determined by the symmetry of the crystal and the propagation direction. The pure transverse and longitudinal modes can only be found in [100] and [001] directions in a hexagonal crystal; the sound propagating modes in other directions are the quasi-transverse or quasi-longitudinal waves. In the primary directions, the sound velocities in a hexagonal crystal can be expressed by

(1) $$\begin{array}{l} {}[100]:[100]v_{l}=\sqrt{(C_{11}-C_{12})/2\rho },[010]v_{t1}=\sqrt{C_{11}/\rho },[001]v_{t2}=\sqrt{C_{44}/\rho }\\ {}[001]:[001]v_{l}=\sqrt{C_{33}/\rho },[100]v_{t1}=\sqrt{C_{44}/\rho },[010]v_{t2}=\sqrt{C_{44}/\rho } \end{array}$$

where *v_t_*_1_ and *v_t_*_2_ refer to the first transverse mode and the second transverse mode, respectively. The calculated sound velocities along the primary directions are listed in Table 5. For *h*-GeCN, in the [001] propagation direction with polarization direction [001], the longitudinal sound velocities *v_l_* have the greatest sound velocity at 0 GPa. The smallest sound velocities result along the [001] propagation direction, with polarization directions [100]*v_t_*_1_, [010]*v_t_*_2_; and along the [100] propagation direction, with polarization direction [001]*v_t_*_2_. From Table 5, it can be seen that for the sound waves along different propagation directions, the sound velocities have different values. In a sense, the *h*-GeCN is anisotropic. In addition, the sound waves along different propagation directions increase with increasing pressure from 0 GPa to 15 GPa, while the [001] propagation direction with polarization directions [100]*v_t_*_1_, [010]*v_t_*_2_, and the [100] propagation direction with polarization direction [100]*v_l_* decrease from 15 GPa to 20 GPa. Because the difference between *C*_11_ and *C*_12_ is smaller, *C*_44_ decreases from 15 GPa to 20 GPa.

**Figure 4:**
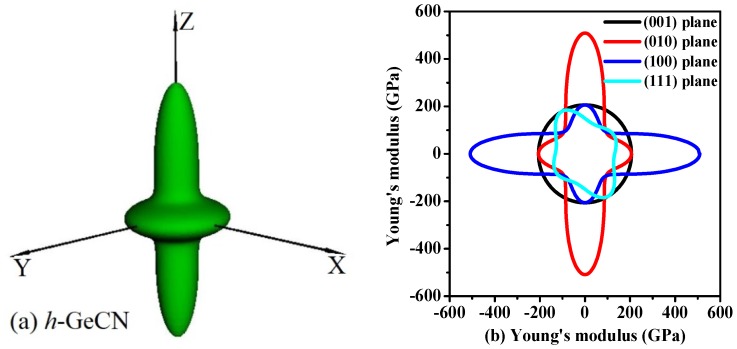
The surface construction of Young’s modulus for *h*-GeCN (**a**); and the 2D representation of Young’s modulus for *h*-GeCN in the (001) plane, (010) plane, (100) plane, and (111) plane (**b**). The black, red, blue, and cyan lines represent the Young’s modulus of *t*-Si_3_N_4_, *t*-Si_2_GeN_4_, *t*-SiGe_2_N_4_, and *t*-Ge_3_N_4_, respectively. All units are in GPa.

The Young’s modulus of the *h*-GeCN also exhibits anisotropy. The directional dependence of Young’s modulus for *h*-GeCN and two-dimensional (2D) representations of Young’s modulus in the (001), (010), (100), and (111) planes for *h*-GeCN are illustrated in Figure 4a,b, respectively. From Figure 4a, the shape of the three-dimensional representations of the Young’s modulus for *h*-GeCN is similar to a gyroscope with the middle width and the two ends sharp. The two-dimensional representations of Young’s modulus for *h*-GeCN are unfolding figures that cut along the (001), (010), (100), and (111) planes, where black, red, blue, and cyan lines represent the (001), (010), (100), and (111) planes, respectively. The figure obtained along the (001) plane is a circle, and the two figures along the (010) and (100) planes are the same, for a gyroscope plane shape, while the (111) plane is an irregular figure. What is more interesting is that the maximum value (509 GPa) of Young’s modulus for *h*-GeCN occurred at the *Z*-axis, but the minimum value (130 GPa) of the Young’s modulus for *h*-GeCN occurred at *θ* = 0.87, *φ* = 5.08 (more details see [55,56,57]). Regardless of the three-dimensional figure of the Young’s modulus and the ratio of the maximum to the minimum (*E*_max_/*E*_min_ = 509/130 = 3.92), it is more than that of *t*-GeCN (*E*_max_/*E*_min_ = 2.49) [15], so the *h*-GeCN has larger anisotropy. In the (010) and (100) planes, the ratio *E*_max_/*E*_min_ = 509/130 = 3.92; this is the largest ratio of elastic anisotropy in the Young’s modulus among these planes. In the (001) plane, the maximal and minimal values of Young’s modulus are both 206 GPa, so the ratio of Young’s modulus in the (001) plane is *E*_max_/*E*_min_ = 206/206 = 1.00; therefore, the Young’s modulus exhibits isotropy in the (001) plane, and it is the smallest elastic anisotropy in Young’s modulus among these planes.

### 3.4. Electronic Properties

**Figure 5:**
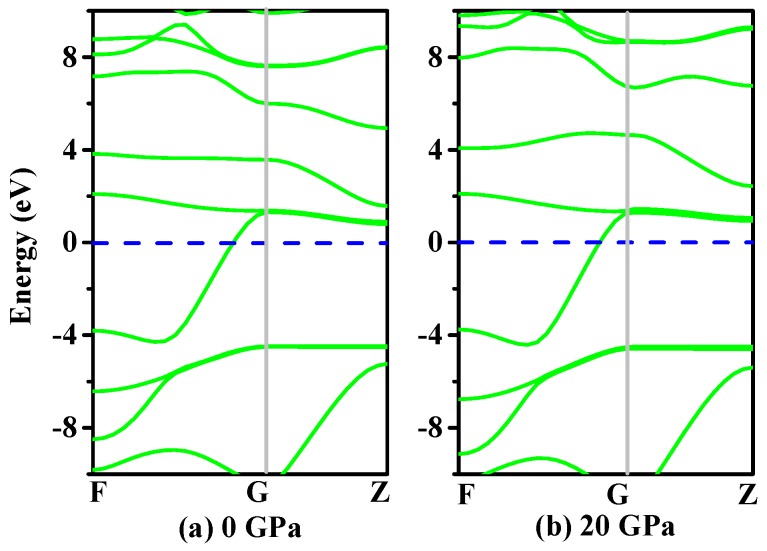
The band structures of *h*-GeCN at 0 GPa (**a**) and 20 GPa (**b**).

It is well known that the electronic structure determines the fundamental physical and chemical properties of materials [35]. The electronic structures of *h*-GeCN (using the rhombohedral cell) under 0 GPa and 20 GPa are shown in Figure 5. From Figure 5, we can see that *h*-GeCN exhibits metallicity. From 0 GPa to 20 GPa, the electronic structures of *h*-GeCN (using the rhombohedral cell) are almost unchanged. The Fermi energy level is the highest level of electrons full of electrons in a solid energy band when the temperature is absolute zero. The Fermi level of *h*-GeCN at 0 GPa is 3.50 eV, while the Fermi level of *h*-GeCN is 4.30 eV when the pressure is under 20 GPa.

## 4. Conclusions

In this work, the structural, elastic, elastic anisotropic, and electronic properties of *h*-GeCN in the *R*3*m* space group were investigated utilizing first-principle calculations. The mechanical and dynamical stability of *h*-GeCN were proved by elastic constants and phonon spectra. The ratio *B*/*G* and Poisson’s ratio *v* of the *h*-GeCN are less than 1.75 and 0.26, respectively, both of which indicate that the *h*-GeCN is brittle. For *h*-GeCN, from brittleness to ductility, the transformation pressures are 5.56 GPa and 5.63 GPa for *B*/*G* and Poisson’s ratio *v*, respectively. At ambient pressure, the Debye temperature of *h*-GeCN is 506 K—smaller than that of *t*-GeCN. The calculated Young’s modulus along all directions and in the primary planes, and the sound velocities along the primary directions of *h*-GeCN, exhibit greater elastic anisotropy. A three-dimensional figure of the Young’s modulus was presented, and the ratio of the maximum to the minimum (*E*_max_/*E*_min_ = 509/130 = 3.92) is greater than that of *t*-GeCN (*E*_max_/*E*_min_ = 2.49). In addition, the band structure reveals that the *h*-GeCN is metallic.

## Figures and Tables

**Table 1:** The calculated lattice parameters (in Å), cell volume (in Å^3^), and density (in g/cm^3^) of hexagonal germanium carbonitride (*h*-GeCN) and tetragonal structure germanium carbonitride (*t*-GeCN).

Material	Methods	Pressure	*a*	*c*	*V*	*ρ*
*h*-GeCN	GGA	0	3.165	10.701	92.819	5.292
		5	3.122	10.627	89.687	5.477
		10	3.087	10.570	87.207	5.633
		15	3.056	10.523	85.100	5.772
		20	3.029	10.480	83.270	5.899
*t*-GeCN	GGA	0	4.326	7.046	131.861	4.967
		0 ^1^	4.323	7.037	131.490	4.981
	LDA	0	4.216	6.993	124.298	5.269
		0 ^1^	4.205	6.892	121.836	5.376

^1^ Ref. [15]. GGA, generalized gradient approximation. LDA, local density approximation.

**Table 2:** The calculated elastic constants (in GPa) of *h*-GeCN and *t*-GeCN.

Material	Pressure	*C*_11_	*C*_12_	*C*_13_	*C*_14_	*C*_24_	*C*_33_	*C*_44_	*C*_56_	*C*_66_
*h*-GeCN	0	232	77	21	4	−4	512	42	4	78
	5	282	95	48	−7	7	586	60	−7	93
	10	329	106	68	−20	20	644	65	−20	112
	15	361	126	91	−27	27	694	68	−27	117
	20	380	158	109	−33	33	741	59	−33	111
*t*-GeCN	0 ^1^	263	143	94	-	-	492	151	-	167

^1^ Ref. [15].

**Table 3:** The calculated elastic modulus (in GPa) of *h*-GeCN.

Material	Pressure	*B*	*G*	*B/G*	*E*	*v*	*A* ^U^
*h*-GeCN	0	130	77	1.688	193	0.253	2.112
	5	165	95	1.737	239	0.258	1.463
	10	193	104	1.856	264	0.272	1.722
	15	219	107	2.047	276	0.290	1.950
	20	244	98	2.490	259	0.323	3.021
*t*-GeCN	0	183	129	1.42	313	0.210	0.925

**Table 4:** The density (*ρ* in g/cm^3^), sound velocity (*v_l_*, *v_t_*, *v_m_*, in m/s), and Debye temperature (*Θ_D_* in K) of *h*-GeCN under pressure.

Materials	Pressure	*v_p_*	*v_s_*	*v_m_*	*Θ_D_*
*h*-GeCN	0	6631	3814	4236	506
	5	7297	4165	4628	559
	10	7673	4297	4783	583
	15	7916	4306	4803	590
	20	7970	4076	4566	565

**Table 5:** The sound velocities along different directions of *h*-GeCN at different pressures.

Material	*P*	[100]			[001]		
		[100]*v_l_*	[010]*v_t_*_1_	[001]*v_t_*_2_	[001]*v_l_*	[100]*v_t_*_1_	[010]*v_t_*_2_
*h*-GeCN	0	3827	4682	1992	6955	1992	1992
	5	4132	5074	2340	7314	2340	2340
	10	4449	5404	2402	7561	2402	2402
	15	4512	5592	2427	7754	2427	2427
	20	4338	5675	2236	7925	2236	2236
